# The effect of civil money penalties on the financial performance of nursing homes

**DOI:** 10.1093/geroni/igag002

**Published:** 2026-01-14

**Authors:** Patrick Schumacher

**Affiliations:** Rockefeller College of Public Affairs & Policy, University at Albany—State University of New York, Albany, New York, United States

**Keywords:** Regulatory enforcement, Profitability, Financial stability, Panel data analysis

## Abstract

**Background and Objectives:**

Civil money penalties (CMPs) are an important tool for holding nursing homes accountable for regulatory noncompliance. These fines can range from a few thousand to hundreds of thousands of dollars. Meanwhile, many nursing homes operate under financial strain, which has been linked to compromised quality of care. This study assesses the impact of CMPs on 2 indicators of financial performance: net income margin and short-term debt.

**Research Design and Methods:**

These indicators were regressed on penalty size using a 2-way fixed effects model, encompassing 87,249 facility-year observations from U.S. nursing homes from 2012 to 2019. The dollar amount of penalties was expressed in equivalent days of operating expenses to account for variation in facility sizes, and the analysis controlled for the nursing home characteristics and market conditions.

**Results:**

A negative relationship was found between the size of penalties and net income margin. Penalties equivalent to less than 1 day of expenses were associated with a 0.86 percentage-point decrease in net-income margin; 1-day penalties with a 1.48-point decline; 2-day penalties with a 2.49-point reduction; and those equivalent to 3+ days corresponded to a 3.56-point decline. Effects on short-term debt were minimal, with only modest increases seen.

**Discussion and Implications:**

These findings raise a concern for regulators that CMPs, if not carefully calibrated, have the potential to cross the boundary from deterring violations to jeopardizing the financial stability of nursing homes. If a facility struggles to absorb the cost, a CMP could inadvertently affect the care provided to residents.

Innovation and Translational Significance: This study investigated the impact of civil money penalties on the financial health of nursing homes. It uses national data from 2012 to 2019 to compare changes in profitability and short-term debt across facilities with penalties of varying sizes. The findings indicate that large penalties are associated with lower profit margins. This matters because previous research has found an association between weaker financial performance and a higher risk of closure and regulatory violations. The study’s results can help inform policies to make sure that penalties are effective without unintentionally weakening the financial stability of nursing homes.

The Centers for Medicare and Medicaid Services (CMS) conducts regular inspections of nursing homes, in partnership with state survey agencies, to monitor nursing homes’ compliance with federal regulations (Centers for Medicare and Medicaid Services [CMS], 2023). Facilities found in non-compliance may receive a sanction, also known as a remedy, from CMS. The most common type of remedy is the civil money penalty (CMP). A CMP can range from a few thousand to hundreds of thousands of dollars, depending on the scope and severity of the violations identified. The CMP can be imposed on a per-instance basis, a single fine, or a per-diem basis, in which the amount of the penalty accumulates for each day of non-compliance. Records from CMS show that the agency imposed a total of $202.9 million in penalties on nursing homes in 2024 (CMS, 2025). Per diem penalties averaged $57,567, and per instance penalties averaged $11,852. These financial penalties are designed to hold nursing homes accountable and are a valuable tool for protecting residents from abuse, substandard care, and neglect (Miles, 2021).

Deterrence theory holds that penalties curtail undesirable behavior by imposing or threatening punishment (Becker, 1968). For nursing homes, the first point of impact of a CMP is financial performance. An important yet unexplored area of research is the investigation of how CMPs impact the financial health and stability of nursing homes. Prior studies indicate that many nursing homes in the United States operate from a vulnerable financial position. One study found that 21% of nursing homes in the United States operated without a profit in 2019, after excluding disallowed costs and depreciation from net income (Harrington et al., 2023). The COVID-19 pandemic exacerbated these financial challenges. A survey of nursing homes in August 2020 by the American Health Care Association and the National Center for Assisted Living found that over half of respondents reported operating at a loss (American Health Care Association, 2020). Another study found that operating margins for nursing homes dropped from approximately 2.7%, on average, before the pandemic to −3.0% during the pandemic (Orewa et al., 2024). The study also identified a 7-percentage-point decline in occupancy during the same period.

The operation of a nursing home is resource intensive. Salaries for staff represent a major expenditure, particularly as nursing homes increasingly rely on costly labor from temporary workers supplied by staffing agencies (Bowblis et al., 2024a). Profit margins also come under pressure because the reimbursement for services from the Medicaid Program is low. According to KFF, Medicaid is the primary payer for 63% of nursing home residents (Chidambaram & Burns, 2024). The Medicaid program, however, sometimes reimburses nursing homes at less than the cost of care for services. A federal report estimated that, on average, Medicaid reimbursements covered only 82% of the actual costs incurred by nursing homes for Medicaid residents in 2019 (Bowblis et al., 2024b). In other words, for every dollar that nursing homes spent on care for residents covered by Medicaid, they faced an average shortfall of about 18 cents.

Financial challenges experienced by nursing homes are concerning, given that studies have linked poor financial performance to substandard conditions for residents. One study found that nursing homes with low profit margins had a higher likelihood of receiving repeated citations related to infection control than facilities with higher profit margins (Sharma & Xu, 2021). Another study of nursing homes in Florida found that facilities in the lowest quartile of financial performance, based on net-income margin and operating margin, were more likely to have residents with worse outcomes, such as higher rates of urinary catheterization (Oetjen et al., 2011). Declines in net-income margin have also been linked to the closure of nursing homes. A study of nursing homes in California found that facilities with a decline of 5-percentage points or more in their net income margin were more than twice as likely to close over 5 years than were those with a stronger financial performance (Kitchener et al., 2004).

Building on prior research, this study examines the impact of civil-money penalties on two indicators of the financial health of a nursing home: (1) net-income margin and (2) short-term debt. Net-income margin reflects overall profitability and serves as an indicator of financial health. Short-term debt captures the extent of a facility’s financial obligations due within 12 months. This study examines whether large penalties are associated with a decrease in net-income margin and an increase in short-term debt. The study is conducted on a sample of nursing homes in the United States from 2012 to 2019 using a two-way fixed effects model. This assessment of how penalties affect financial viability is an important step toward understanding the potential downstream effects that could influence the quality of care for residents.

## Method

### Data sources

The study uses two sources of data from the CMS: (1) Skilled Nursing Facility (SNF) Cost Reports and (2) the Quality and Certification Oversight Reports (QCOR) database. The SNF Cost Reports are financial statements that CMS requires nursing homes to submit annually (42 CFR § 413.20) (CMS, n.d.). These reports provide detailed information on the financial health of facilities, including operating costs, revenues, and liabilities. The reports are the only publicly available and comprehensive source of information on the finances of U.S. nursing homes, and as such, they have been used widely in research, including to identify nursing homes at risk of closure (Lord et al., 2020), to assess the financial impact of COVID-19 (Orewa et al., 2024), and to examine links between financial performance and quality of care (Weech-Maldonado et al., 2019). Data on the timing and amount of civil money penalties (CMPs) were downloaded from the QCOR database, a federal repository of information on regulatory activity at nursing homes (CMS, 2025).

Characteristics of facilities, including variables related to the number of residents, staffing, and complexity of care, were obtained from the Long-term Care Focus (LTCFocus) dataset curated by Brown University (Brown University, n.d.). The LTCFocus dataset aggregates data from multiple sources, including the Minimum Data Set, and is frequently used in the literature. Finally, market-level characteristics for the county in which each nursing home was located were incorporated using data from the American Community Survey (for median household income) (U.S. Census, n.d.) and the Bureau of Labor Statistics (for county-level unemployment rates) (U.S. Bureau of Labor Statistics, n.d.-a).

### Study population

The initial dataset included 15,238 nursing homes in the United States that submitted SNF Cost Reports between 2011 and 2019. The analysis was limited to data through 2019 to avoid disruptions to data caused by the COVID-19 pandemic in later years. Nursing homes were excluded if they were operated by the government (county, state, or federal) or based in a hospital (*n *= 1,479) because these types of facilities have different accounting structures and function differently from for-profit and non-profit nursing homes and are commonly excluded from studies that use SNF Cost Reports. Facilities located in Puerto Rico were also excluded (*n *= 6).

Several steps were taken to prepare the dataset for analysis. Observations with negative values for operating expenses, liabilities, or revenue were removed. Next, financial variables were winsorized at the 1st and 99th percentiles to limit the influence of extreme values. This method replaces values below the 1st percentile with the value at the 1st percentile and replaces values above the 99th percentile with the value at the 99th percentile (Adams et al., 2019). After winsorization, observations with a net-income margin that exceeded an absolute value of 60% were excluded. Margins outside this range were considered implausible, consistent with the approach taken by Harrington et al. (2023). Similarly, observations were excluded if total current liabilities exceeded 300% of net-patient revenue. After applying these criteria, 403 facilities were removed because they had no valid reports remaining. All financial variables were adjusted to the value of the dollar in 2019 using the Consumer Price Index from the U.S. Bureau of Labor Statistics (n.d.-b).

In addition, facilities were excluded if they lacked at least three years of data with non-missing observations in all variables. This criterion was imposed because models using fixed effects and panel data rely on observing within-facility variation over time to identify effects; requiring a minimum of 3 years ensures that at least two periods of change can be analyzed within each facility. Facilities failing to meet this threshold were excluded (*n *= 1,131). Finally, observations from 2011 were removed. Reports from 2011 were obtained solely to construct 1-year lags on the dependent variables; they were not included in the analysis because records from 2011 cannot have a prior-year lag. The final dataset consisted of 12,219 nursing homes with 87,249 facility-year observations.

### Dependent variables

Two financial indicators were used as the dependent variable to capture different facets of the financial health of a nursing home. Net-income margin, a measure of profitability, was calculated by dividing net income by the sum of net-patient revenue and total other income (Equation 1). The second dependent variable, short-term debt, was scaled to the size of each facility by dividing total current liabilities by net-patient revenue and multiplying by 100 (Equation 2). Additional details on the specific line items from the SNF Cost Reports corresponding to each variable are shown in Supplementary Table 1 (see online supplementary material).


(1) $$\mathrm{Net}\;\mathrm{income}\;\mathrm{margin}=\;\frac{\mathrm{Net}\;\mathrm{income}}{\mathrm{Net}\;\mathrm{patient}\;\mathrm{revenue}+\mathrm{total}\;\mathrm{other}\;\mathrm{income}}*100$$

(2) $$\mathrm{Short}\;\mathrm{term}\;\mathrm{debt}\;\mathrm{adjusted}=\;\frac{\mathrm{Total}\;\mathrm{current}\;\mathrm{liabilities}}{\mathrm{Net}\;\mathrm{Patient}\;\mathrm{revenue}}*100$$

### Independent variable

The primary independent variable was the aggregate dollar amount of all penalties, if any, imposed on a nursing home each year. The dollar amount of penalties was expressed relative to daily operating expenses to standardize the variable and facilitate more straightforward interpretation. In Equation 3, the numerator represents the annual sum of all CMPs imposed on nursing home *i*. The denominator reflects the yearly operating expenses of the facility, divided by 365 days to convert the value into daily operating expenses.


(3) $${\text{Penalty}\;\text{Impact}}_{i}(\text{in}\;\text{days})=\frac{\sum {\text{Penalty}\;\text{Amount}}_{i}}{\left(\frac{{\text{Operating}\;\text{Expenses}}_{i}}{365}\right)}$$

After calculating the impact of the penalties in days, dummy variables were created to categorize the impact of the penalties in days of operating expenses. The categories are defined as follows: “no penalty” (reference category), “less than one day” (penalty greater than zero but less than one day of operating expenses), “one day” (penalty equivalent to at least one but less than 2 days of expenses), “two days” (penalty equivalent to at least two but less than three days of expenses), and “greater than three days” (penalty equivalent to three or more days of operating expenses). Each was coded as 1 if the condition was met, and 0 if otherwise. These categories were chosen based on the distribution of penalties observed in the data, to capture potential threshold effects, and to improve interpretability. The distribution of penalties across these categories is presented in the Results section.

### Control variables

The control variables consisted of annual facility-level characteristics commonly controlled for in the literature. The average daily number of residents represented the size of the facility. The occupancy rate, a related but distinct measure, captured the share of occupied beds in the facility. The percentage of residents covered by Medicaid was included to control for payer mix, as Medicaid, Medicare, and private-pay sources reimburse services at different rates. The study also controlled for the Resource Utilization Group Nursing Case Mix Index, which is a measure that reflects the average intensity of care required by residents. In addition, the study controlled for market-level characteristics that could influence the financial health of a facility. The Herfindahl–Hirschman Index (HHI), which ranges from 0 to 1, was used to control for market concentration. Facilities in more concentrated markets (HHI closer to 1) have less competition, meaning that they may have greater pricing power or more stable occupancy, which could affect profitability. The HHI was calculated as the sum of the squared market shares of all nursing homes in the county, with market share defined as the number of beds in a facility divided by the total number of beds in the county. Median household income and unemployment rate were included to capture economic conditions in the county where a nursing home operates. The analysis also included lagged dependent variables to account for the fact that past financial performance may influence future financial performance.

### Statistical analysis

The financial indicators were regressed on the size of penalties, represented as days of operating expense, while controlling for facility characteristics and local economic conditions. Two-way fixed effects for both facility (unit) and year were used with robust standard errors clustered at the facility level. Within-facility changes constitute the variation used in the two-way fixed-effects regressions. Among those facility‐year observations that experienced a change in penalty status, 8,456 changed from no penalty to any penalty, 7,628 changed from any penalty to no penalty, and 1,624 moved between penalty categories (for example, from a 1-day penalty in *t-1* to a 2-day penalty in *t*). In addition, descriptive statistics were calculated for all variables used in the analysis. All statistical analyses were conducted using R (Version 4.4.0; R Core Team, 2024). The *plm* package was used for the panel regression (Version 2.6-3; Croissant and Millo, 2008).

## Results

Summary statistics for all facility-year observations are included in Table 1. The average net-income margin was 0.20% (*SD* = 9.50). In other words, on average, facilities retained just 20 cents in profit for every $100 of total revenue. Short-term debt averaged 23% of revenue (*SD* = 34.16). Most facility-year observations had no penalty (85%). Those that had a penalty comprised the following shares: equivalent to less than 1 day of operating expenses (9%); 1 day (2%); 2 days (1%); and greater than or equal to 3 days (2%). The average daily census for nursing homes was 89.96 residents (*SD* = 50.13); the average rate of occupancy was 82.05%. The average Resource Utilization Group Nursing Case Mix, representing the complexity of care, was 1.17. The average percentage of residents on Medicaid was 60.25%. The mean staff-to-resident ratio was 3.54 hr per resident per day. Facilities operated in counties with an average unemployment rate of 5.53% and an average median income of $58,636. The HHI, which represents market concentration, averaged 0.19.

**Table 1:** Summary statistics for variables used in analysis (*n *= 87,249 observations).

Variable	Mean	*SD*	Minimum	Maximum
**Net income margin**	0.20	9.50	−59.92	58.09
**Short-term debt (% of revenue)**	23.00	34.16	0.13	299.36
**Penalty^a^**				
** No penalty**	0.85	0.36	0.00	1.00
** <1 day**	0.09	0.29	0.00	1.00
** 1 day**	0.02	0.15	0.00	1.00
** 2 days**	0.01	0.10	0.00	1.00
** ≥3 days**	0.02	0.15	0.00	1.00
**Average daily census**	89.96	50.13	10.03	770.18
**Occupancy rate**	82.05	13.74	5.94	100.00
**Case Mix Index**	1.17	0.15	0.58	3.39
**Percent Medicaid residents**	60.25	21.70	0.00	100.00
**Direct care hours per patient day**	3.54	0.84	0.05	20.73
**Herfindahl–Hirschman Index**	0.19	0.23	0.00	1.00
**Median household income**	58,636	15,746	20,092	142,299
**Unemployment rate**	5.53	2.13	1.07	27.72

*Note*. *SD* = standard deviation.

a Penalty variables represent the share of facility-year observations in which a facility received each type of penalty during the year.

**Figure 1:**
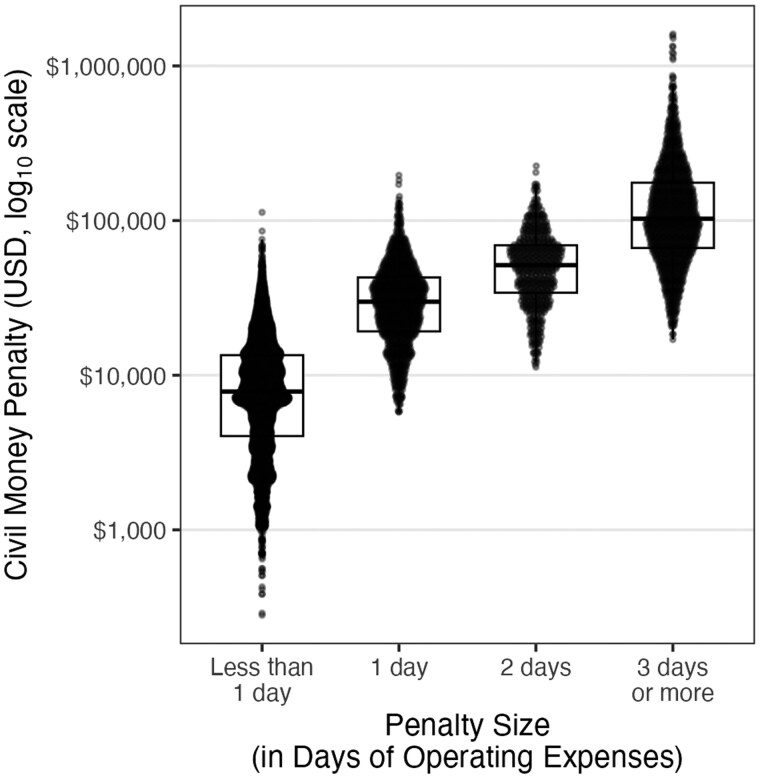
Civil money penalties by category of relative size (in days of operating expenses). Note. Figure 1 shows the distribution of civil money penalties in dollars by categories reflecting the size of penalties, expressed in days of operating expenses. Categories on the x-axis reflect the size of each penalty relative to a facility’s average daily operating cost (e.g., “1 day” indicates a penalty equal to one day’s expenses). The y-axis is plotted on a base-10 logarithmic scale but labeled in raw dollars for readability. Boxplots show the median, interquartile range, and 1.5 × IQR whiskers; individual points display the distribution of the dollar amount of penalties within each category.

Table 2 presents the distribution of CMPs imposed on nursing homes between January 2012 and December 2019. The share of nursing homes receiving at least one CMP increased from 11.0% in 2012 to 20.7% in 2017, before slightly declining. The mean annual penalty rose from $26,085 in 2012 to a peak of $50,185 in 2016, with the largest penalty exceeding $1.6 million in that year. Figure 1 shows the distribution of CMPs by dollar amount, categorized by the size of the penalty, expressed as equivalent days of operating expenses. These categories represent the size of each penalty compared to the facility’s average daily operating cost. For example, a penalty in the “1 day” category indicates that the penalty amount was approximately equal to the facility’s average expenses for 1 day. Median penalties in each category were $7,962 (“Less than 1 day”), $29,958 (“1 day”), $52,009 (“2 days”), and $109,694 (“3 days or more”).

**Table 2.** Summary of penalties imposed each year on nursing homes in the study population.

Year	Total homes	Homes with penalty	% with penalty	Mean penalty (USD)	*SD* of penalty (USD)	Min penalty (USD)	Max penalty (USD)
**2012**	10,035	1,099	11.0%	$26,085.19	$58,312	$289.51	$623,723
**2013**	11,297	1,328	11.8%	$30,299.65	$66,258	$384.11	$957,959
**2014**	11,304	1,336	11.8%	$35,652.43	$79,865	$245.68	$1,344,074
**2015**	11,139	1,409	12.6%	$38,436.91	$83,320	$105.17	$1,322,310
**2016**	11,027	1,830	16.6%	$50,185.16	$102,578	$207.72	$1,607,106
**2017**	11,125	2,298	20.7%	$40,614.41	$90,282	$620.32	$1,565,853
**2018**	10,649	1,843	17.3%	$41,840.82	$79,886	$412.34	$1,281,169
**2019**	10,673	1,878	17.6%	$43,494.65	$69,418	$325.00	$830,579

*Note*. *SD* = standard deviation.

Table 3 presents two-way fixed effects estimates of CMPs (in days of operating expenses) on net-income margin. Compared to no penalty, penalties under one day were associated with a 0.86 percentage-point reduction in net-income margin (*p < *.01), 1-day penalties with a 1.48-point reduction (*p < *.01), 2-day penalties with a 2.49-point reduction (*p < *.01), and penalties of three or more days with a 3.56-point reduction (*p < *.01). Associations with short-term debt were more limited: only 1-day (β  =  1.07, *p < *.05) and three-plus-day penalties (β  =  1.41, *p < *.01) significantly increased debt as a percentage of revenue. In practical terms, a 1-day CMP raised short-term debt by 1.07 percentage points, which for a nursing home with $500,000 in revenue, equated to approximately $5,350 increase in current liabilities.

**Table 3.** Fixed-effects analysis of civil money penalties on net-income margin and short-term debt.

Variable	(1) Net income margin		(2) Short-term debt (% of revenue)	
**Penalty (reference: no penalty)**				
** <1 day**	−0.858***	(0.089)	−0.081	−0.081
** 1 day**	−1.480***	(0.191)	1.070**	1.070**
** 2 days**	−2.490***	(0.336)	1.480	1.480
** ≥3 days**	−3.560***	(0.235)	1.410***	1.410***
**Average daily census**	0.137***	(0.007)	−0.130***	−0.130***
**Occupancy percent**	0.104***	(0.006)	−0.088***	−0.088***
**Case Mix Index**	5.240***	(0.479)	−5.570***	−5.570***
**Percent Medicaid**	−0.028***	(0.003)	0.023***	0.023***
**Direct care hours per patient day**	−0.419***	(0.059)	0.265	0.265
**Herfindahl–Hirschman Index**	0.799	(1.460)	−4.910*	−4.910*
**Median income (logged)**	−2.840***	(1.040)	2.920	2.920
**Unemployment rate**	0.040	(0.042)	−0.357***	−0.357***
**Lagged net margin (1 year)**	0.186***	(0.006)		
**Lagged debt adjusted (1 year)**			0.437***	0.437***
**Observations**	87,249		87,249	
***R* ^2^**	0.113		0.165	
***F* statistic (df = 13; 75010)**	738.000***		1,139.000***	

*Note.* Coefficients with standard errors in parentheses are reported.

* *p < *.1.

** *p < *.05.

*** *p < *.01.

## Discussion and implications

This study assessed the effect of civil money penalties on two financial indicators for nursing homes: net-income margin and short-term debt. The results suggest a negative association between the size of CMPs and net-income margin. This negative effect intensified as penalties represented a larger share of daily operating expenses. Penalties equivalent to less than 1 day of operating expenses were associated with a 0.86 percentage-point decline in net-income margin, 1-day penalties with a 1.48-point decline, 2-day penalties with a 2.49-point decline, and penalties of three or more days with a 3.56-point decline. These results indicate that CMPs erode profitability. The decrease in net-income margin may also reflect additional legal, administrative, or reputational costs beyond the face value of the fine.

In contrast to the results for net-income margin, the analysis showed a more limited relationship between the size of penalties and short-term debt (expressed as a percentage of revenue). Statistically significant associations were observed only for penalties equivalent to one day of expenses and to penalties equivalent to three or more days; both penalty equivalencies were associated with only modest increases in short-term debt as a percentage of revenue. These findings suggest that while some increase in debt is observable following CMPs, the overall financial response does not appear to involve reliance on short-term liabilities. This limited effect on short-term debt might also stem from facilities using financial strategies not reflected in Cost Reports. For example, they may rely on lines of credit that are repaid within the fiscal year and, therefore, are not recorded as year-end liabilities.

The effect of penalties on net-income margin may offer insight for policymakers. Previous studies have found that a decline of more than 5% in net-income margin puts nursing homes at risk of closure (Kitchener et al., 2004). Closure of a facility has been associated with negative outcomes for residents, including heightened risk for falls and psychological stress after relocation (Friedman et al., 1995; Weaver et al., 2020). However, other research suggests that although closures can have deleterious short-term effects, they may lead to improved outcomes for residents in the long-term if they relocate to higher-quality facilities (Olenski, 2023). Low net-income margin has also been associated with worse care, with one study finding that facilities with low net-income margin are more likely to have violations related to control of infection than are facilities with a higher net-income margin (Sharma & Xu, 2021). Because declining margins may increase the risk of closure and violations, regulators should consider a more consistent framework for incorporating financial conditions when determining the size of a penalty. Policymakers should also encourage proactive communication between regulators and administrators before imposing a penalty. This could help both parties assess the ramifications of a penalty and identify strategies for maintaining fiscal stability.

The finding of a negative association between the size of CMPs and net-income margin should not be misconstrued as indicating that CMS should broadly reduce the size of CMPs. A penalty is intended to function as a deterrent because it negatively impacts the financial health of a facility; a reduction in net income-margin is an expected consequence of a penalty necessary to discourage regulatory non-compliance. Rather, this study raises a concern for regulators about the potential for penalties to cross the boundary from acting as a deterrent to adversely affecting the financial stability of nursing homes. This study does not identify a tipping point between deterrence and destabilizing financial harm, but this topic could motivate future research. Future research could also consider employing qualitative methods to give a deeper understanding of the impact of penalties. Interviews or focus groups, for example, could provide insights into how administrators of nursing homes perceive the effect of CMPs on their operations. There is also little empirical evidence on whether the size of civil money penalties leads to ensuing improvements in quality of care. Future research on this topic could clarify the effectiveness of CMPs as a regulatory tool.

The results should be viewed with consideration of the following limitations of the study. First, there is a risk of reverse causality in the regression model because regulators may consider the financial condition of nursing homes when assessing a penalty. In this conception, the size of penalties not only affects the size of financial indicators, but the financial indicators also affect the size of penalties. Regulation 42 CFR 488.438(f)(2) states that CMS or the States must consider the financial condition of a facility when determining the amount of a penalty. To what extent regulators factor financial condition into their decisions, or what specific aspects of financial condition they consider, however, is unclear. Second, the data used in this study may not reflect reductions in the size of CMPs from appeals. Nursing homes can appeal a CMP through an informal dispute resolution or a formal administrative appeal (42 CFR 488.331). Our estimates, therefore, may overstate the actual financial impact of CMPs to the extent that penalties are reduced or eliminated through appeals. Third, the data from the SNF Cost Reports are self-reported by nursing homes and unaudited by CMS, potentially making it subject to errors or misreporting (U.S. Government Accounting Office, 2016). The Cost Reports may also not reflect the true profitability of nursing homes that conceal earnings through complex ownership structures (Harrington et al., 2015). Fourth, information on citations, and their associated penalties, are publicly available, and prior research suggests that residents and their families may use this information when choosing between nursing homes. This may make it difficult to disentangle the financial effect of a CMP from consumer response (Perraillon et al., 2019).

In addition, this study does not consider other types of corrective actions that CMS may take in response to violations, either alone or concurrently with CMPs. Examples include mandating that staff undergo remedial education, appointing an outside monitor, or placing the facility under temporary management. Most relevant to this study, regulators can also deny reimbursement to facilities for services under the Medicare and Medicaid program (referred to as denial of payment for new admissions or DPNA). Assessing the financial impact of DPNA, however, was not feasible due to a lack of data concerning the denial’s duration, the number of affected admissions, and reimbursement rates. As a result, the financial effects observed in this study may be partially influenced by unmeasured, concurrent remedies, such as DPNA.

Finally, this study represents only a first step in understanding how CMPs impact residents’ well-being. The financial indicators examined here, net-income margin and short-term debt, are intermediate outcomes that could affect a facility’s ability to sustain operations and meet the needs of residents. Going forward, research could build on this work by examining whether CMPs serve as a deterrent and how their imposition, or lack thereof, influences outcomes for residents.

## Supplementary Material

igag002_Supplementary_Data

## Data Availability

All data used in this study are publicly available from the Centers for Medicare and Medicaid Services or, upon request, from the author. All analyses were conducted using R (Version 4.4.0; R Core Team, 2024). This study was not preregistered. This article is based, in part, on the author’s doctoral dissertation.
