# Supplementary material for: The effect of civil money penalties on the financial performance of nursing homes
Source: Innov Aging. 2026 Jan 14;10(3):igag002. doi: 10.1093/geroni/igag002 (PMC12924879; doi:10.1093/geroni/igag002)
Supplement: igag002_Supplementary_Data [file igag002_supplementary_data.docx]

***Innovation in Aging* Supplementary Material: Schumacher. The effect of civil money penalties on the financial performance of nursing homes.**

## **Supplementary Table 1.** Definitions and sources of variables used in the analysis

| **Variable name** | **Definition** | **Source** |
| --- | --- | --- |
| **Dependent variables** | | |
| Net-income margin | The percentage of total revenue remaining after accounting for all expenses. Calculated as Net Income / (Net Patient Revenue + Other Income) * 100. Based on elements *G3-Line-31 / (G3-Line-3 + G3-Line-25) * 100*. | SNF Cost Reports |
| Short-term debt (as a percentage of net patient revenue) | Total current liabilities expressed as a percentage of net patient revenue. Calculated as (Total current liabilities / Net patient revenue) * 100. Based on elements *(G-Line-43-Columns-1 thru 4 / G3-Line-3-Column-1) * 100*. | SNF Cost Reports |
| **Independent variable** | | |
| Penalty impact (categorical) | Dummy variables representing the dollar amount of penalties relative to days of operating expenses. Calculated as Total annual penalties / (Total annual operating expenses / 365). Values categorized into dummy variables: No penalty (ref), <1 day, 1 day (<2), 2 days (<3), ≥3 days. Operating expenses based on element *G3-Line-4-Column-1*. | Calculated using data from The Quality and Certification Oversight Reports Database (QCOR) and CMS SNF Cost Reports |
| **Control variables** | | |
| Occupancy rate | Percentage of available beds filled by residents, based on variable *occpct*. | Long Term Care Focus |
| Average daily census | Average number of residents present per day during the year, based on variable *avg_dailycensus*. | Long Term Care Focus |
| Share of resident’s coverage by Medicaid | The number of facility residents whose primary support was Medicaid, based on variable *paymaid*. | Long Term Care Focus |
| RUG case mix index | The average resource utilization group (RUG) case mix index for all residents in a nursing home. Higher values indicate residents who require more complex care, based on variable *avgrugcmi_mds3*. | Long Term Care Focus |
| Direct Care Hours per Patient Day | Direct-care staff hours per resident day. Based on variable *dchrppd*. | Long Term Care Focus |
| Median household income | Median household income in the county where the nursing home is located. Based on variable *B19013_001*. | US Census Bureau, American Community Survey 5-Year Estimates |
| Unemployment rate | Unemployment rate in the county where the nursing home is located. | US Bureau of Labor Statistics, Local Area Unemployment Statistics |
| Herfindahl-Hirschman Index | Sum of the square of the proportion of beds in each nursing home relative to the total number of beds in the county, for all nursing homes in the county. Based on variable *totbeds*. | Calculated using total beds (totbeds) from Long Term Care Focus |
| Lagged dependent variable | The value of the respective dependent variable (Net income margin, short-term debt) from the previous year (t-1). | SNF Cost Reports |
